# Heralded Spectroscopy Reveals Exciton–Exciton Correlations in Single Colloidal Quantum Dots

**DOI:** 10.1021/acs.nanolett.1c01291

**Published:** 2021-08-16

**Authors:** Gur Lubin, Ron Tenne, Arin Can Ulku, Ivan Michel Antolovic, Samuel Burri, Sean Karg, Venkata Jayasurya Yallapragada, Claudio Bruschini, Edoardo Charbon, Dan Oron

**Affiliations:** † Deptartment of Physics of Complex Systems, Weizmann Institute of Science, Rehovot 7610001, Israel; ‡ Department of Physics and Center for Applied Photonics, University of Konstanz, Konstanz D-78457, Germany; § School of Engineering, École Polytechnique Fédérale de Lausanne (EPFL), Neuchâtel 2002, Switzerland

**Keywords:** quantum dots, multiexcitons, biexciton binding energy, single-particle spectroscopy, SPAD arrays

## Abstract

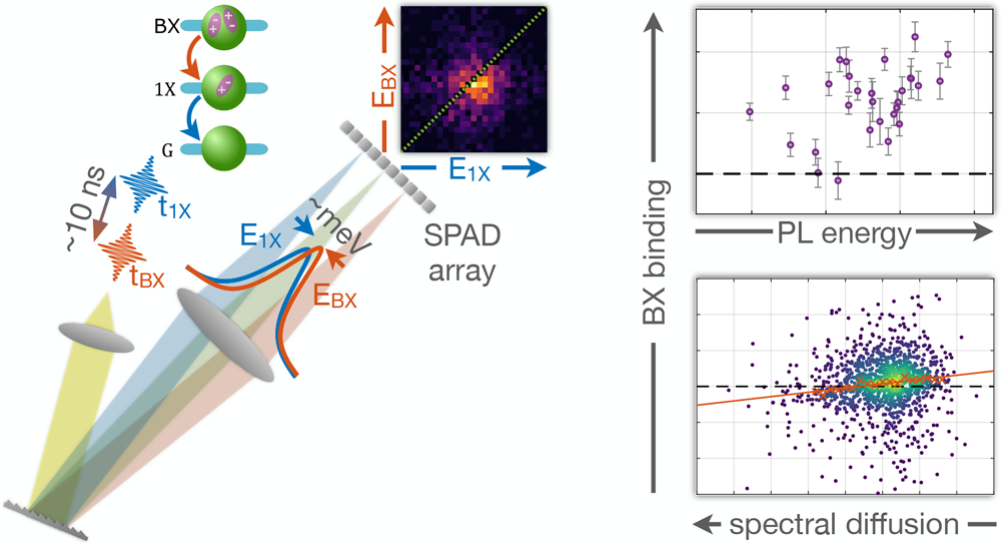

Multiply excited states in semiconductor quantum dots feature intriguing physics and play a crucial role in nanocrystal-based technologies. While photoluminescence provides a natural probe to investigate these states, room-temperature single-particle spectroscopy of their emission has proved elusive due to the temporal and spectral overlap with emission from the singly excited and charged states. Here, we introduce biexciton heralded spectroscopy enabled by a single-photon avalanche diode array based spectrometer. This allows us to directly observe biexciton–exciton emission cascades and measure the biexciton binding energy of single quantum dots at room temperature, even though it is well below the scale of thermal broadening and spectral diffusion. Furthermore, we uncover correlations hitherto masked in ensembles of the biexciton binding energy with both charge-carrier confinement and fluctuations of the local electrostatic potential. Heralded spectroscopy has the potential of greatly extending our understanding of charge-carrier dynamics in multielectron systems and of parallelization of quantum optics protocols.

## Introduction

**Figure 1:**
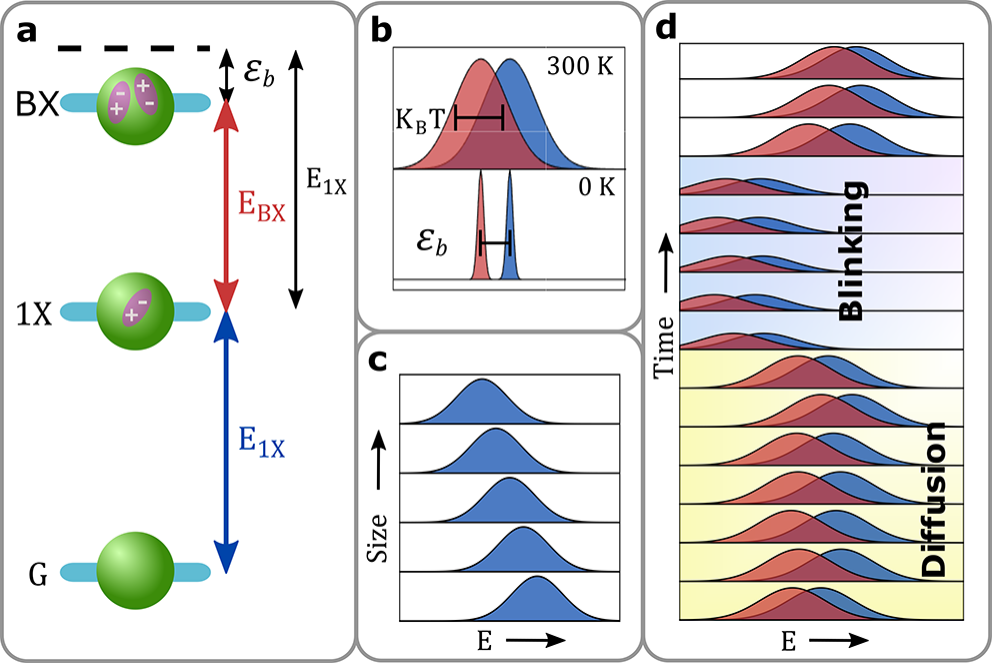
Obstacles in measuring the 1X–BX energy ladder. (a) The energy diagram of the 1X and BX states in a nanoparticle. The energy difference between the BX and 1X states (*E*_BX_) is smaller than the difference between the 1X and ground states (*E*_1X_) by the biexciton binding energy (ε_b_). (b) Schematic of the thermal broadening of spectral lines (intensity normalized for clarity). At room temperature, the 1X-ground (blue) and BX–1X (red) transitions emission lines are broadened to approximately ∼*k*_B_*T* ≈ 26 mev > ε_b_. As a result, the two emission spectra substantially overlap. (c) Schematic dependence of the 1X energy on nanoparticle size. An ensemble measurement (with a ± 5*%* size variance) at room temperature roughly includes a mixture of the depicted spectra. (d) Scheme of spectral drift in the emission lines of a single nanocrystal throughout the measurement time. Apart from the stochastic random walk of the spectral lines (diffusion), discrete spectral jumps typically accompany blinking events.

Over the past three decades, numerous types of semiconductor nanocrystals with varying compositions, shapes, sizes, and structures have been fabricated and studied^1−3^ with some even making their way into mass produced consumer products.^4^ Since the energy of a charge carrier in a nanoconfined solid is quantized, nanocrystals are often referred to as “artificial atoms”. In a further analogy to atomic physics, photoexciting such a quantum dot (QD) generates a hydrogen-like electron–hole state, an exciton, which is typically bound even at room temperature due to the increased Coulomb interaction. However, unlike atoms and molecules, semiconductor nanocrystals include another readily excited manifold of states, multiexcitons, that is, multiple electron–hole pair states.^5^ In the lowest energy multiexcitonic state, the biexciton (BX), a strong exciton–exciton interaction is imposed by the confining potential of the nanoparticle. In the resulting energy ladder of ground, single exciton (1X), and BX states, shown in Figure 1a, the BX state energy is somewhat offset from twice the energy of the 1X state by the BX binding energy (ε_b_).^5^ This binding energy is considered positive (attractive interaction) when *E*_BX_ < *E*_1X_, where *E*_*k*_ is the energy difference between state *k* and the state right beneath it in the ladder.

Cascaded relaxation from the top to the bottom of this ladder can yield a pair of photons; the first around *E*_BX_ and the second around *E*_1X_. Such an emission from self-assembled indium arsenide (InAs) QDs, for example, is a leading candidate for efficiently generating on-demand entangled photon pairs.^6^ On the other hand, avoiding the excitation of the BX state is key for using the same type of QDs as high-purity single-photon sources.^6^ More conventional light-based applications that stand to vastly benefit from the incorporation of colloidally synthesized QDs, such as light emitting diodes (LEDs),^7,8^ lasers,^9,10^ displays,^4^ and photovoltaics,^11^ also require characterization and control of the energy and dynamics of the BX state. For instance, nonradiative Auger recombination often dominates the BX relaxation dynamics.^12^ Therefore, to achieve low threshold lasing from QDs, sophisticated heterostructures have been designed to either increase the BX energy via Coulomb repulsion (avoiding its occupation)^13^ or to reduce the Auger recombination rate.^14^ Furthermore, the Auger-induced low BX quantum yield sets a saturation boundary for the achievable light fluence of nanocrystal-based LEDs.^15^

The above-mentioned interest in the BX–1X ladder inspired substantial spectroscopic efforts to characterize the BX binding energy in multiple material systems such as III–V,^16−18^ II–VI,^19,20^ and lead halide perovskite^21,22^ semiconductor nanocrystals as well as atomically thin films of transition metal dichalcogenides (TMDC).^23^ However, conditions under which the BX–1X ladder can be directly probed are restrictive. While at cryogenic temperatures the very stable and narrow 1X and BX emission lines of a single self-assembled InAs or colloidal cadmium chalcogenide QDs can be discerned,^17,18,24^ in most other cases this was not achieved due to several fundamental limitations. First, at room temperature both emission lines are thermally broadened well-beyond typical BX binding energies (Figure 1b). Spectral features are further broadened in ensemble measurements due to nanoparticle inhomogeneity (Figure 1c). Additional inhomogeneous broadening is caused by spectral fluctuations (Figure 1d). In many nanocrystals, this includes not only the spectral diffusion due to spurious electric fields but also spectral jumps due to other states contributing to fluorescence intermittency.^25,26^

More indirect methods to probe the BX state rely on power-dependent measurement of photoluminescence (PL) (either in a time-resolved or quasi-continuous-wave manner)^20,22,23,27^ or of transient absorption.^21,28^ While careful modeling and analysis of these measurements provided important spectroscopic information, it has often led to large variance in BX binding energies measured in different studies. For example, while some works found very high (∼100 meV) BX binding energies in CsPbX_3_ (X = Cl, Br, I) nanocrystals,^21^ recently a substantially more stringent bound on its magnitude (|ε_b_|< 20 meV) has been reported.^22,29^ Such discrepancies are mainly due to the difficulty in modeling the different mechanisms that affect PL and absorption at high excitation powers, such as charging, oxidation, blinking, and photoinduced damage.^22^ Furthermore, these methods demand disentangling the spectral contributions of the BX and 1X states, which becomes increasingly difficult to perform when these features strongly overlap for small BX binding energies.

While isolating the BX state in the spectral domain alone is a convoluted task, isolating it in the time domain is conceptually simple. A detection of a photon pair emitted from a single nanocrystal (following a short excitation pulse) pinpoints a relaxation cascade^17,18^ first from the BX to the 1X state and then from the 1X to the ground state (Figure 1a). Separating the two consecutive emissions according to their detection times on a nanosecond scale and measuring their respective spectrum facilitates a direct measurement of *E*_BX_ and *E*_1X_ for a single nanoparticle. However, currently available instrumentation does not enable practical implementation of this simple scheme. Namely, a standard spectrometer cannot provide the required temporal resolution since it relies on cameras with a maximal frame rate of ∼10^3^ fps. This can be addressed by replacing the camera with a monolithic single-photon avalanche diode (SPAD) array; a technology that has achieved a considerable performance boost over the past decade.^30,31^ In such a novel spectrometer, termed here spectroSPAD, the spectral information on single photons can be measured and correlated with subnanosecond temporal resolution.

Here, we present the spectroSPAD system and use it to measure spectral correlations in BX–1X emission of single CdSe/CdS/ZnS core/shell/shell QDs with millielectronvolt precision. Thanks to the temporal resolution and high sensitivity of the spectroSPAD, our measurement scheme overcomes all of the aforementioned obstacles (thermal broadening, spectral diffusion, blinking and low BX quantum yield) and easily separates the BX emission from the misleadingly similar trion (“gray”) charged state emission. While for the particular sample under study the average value of the binding energy is ∼6 meV, we disentangle the inhomogeneous size effect and show that its value in individual QDs correlates with the 1X band edge transition energy. Furthermore, we follow the temporal fluctuations of the BX binding energy for a single nanocrystal and find that those correlate with the spectral diffusion of the 1X transition.

## Results and Discussion

### Apparatus

**Figure 2:**
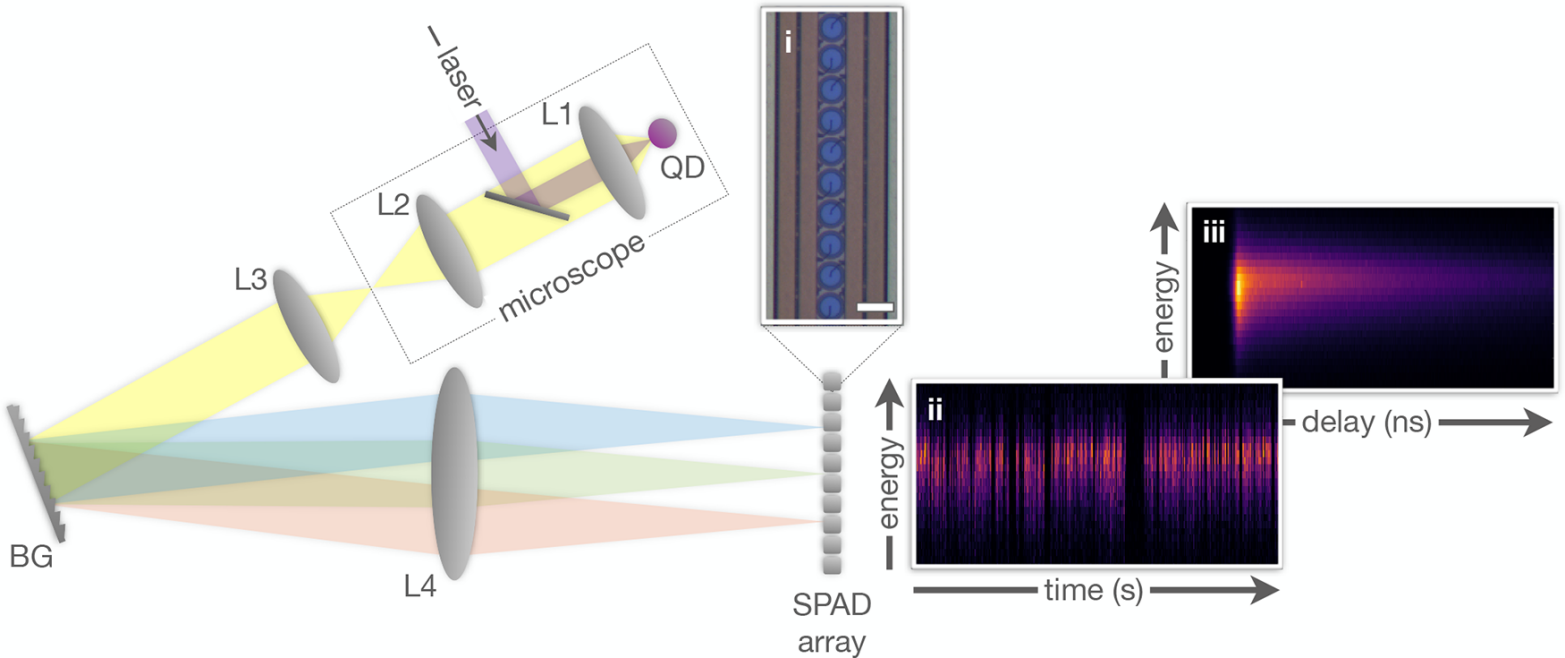
Sketch of the spectroSPAD experimental apparatus. A microscope objective is used to focus a pulsed laser beam on a single QD and collect the emitted fluorescence. At the microscope output, light is passed through a spectrometer setup with a linear SPAD array detector. Inset (i) is an optical image of part of the detector array. Each blue circle represents a single pixel. Scale bar is 30 μm. Insets (ii) and (iii) show two possible analyses of a single nanocrystal spectroSPAD measurement. In (ii), detections are binned according to detection time in the millisecond scale (horizontal) and photon energy (vertical); whereas in (iii) the horizontal axis represents the detection temporal delay from the preceding excitation laser pulse in nanosecond scale. Color-scale corresponds to the number of counts in each temporal-energy bin. Optical elements: objective (L1), tube (L2), collimating (L3), and imaging (L4) lenses; blazed grating (BG).

In recent years, considerable efforts were invested in the design of time-resolved light spectrometers with high sensitivity.^32−36^ As a replacement for the standard CCD camera, different research groups adopted photomultiplier tube (PMT) arrays,^33^ superconducting nanowire single photon detectors (SNSPDs),^35−37^ or SPAD arrays.^32,34,38,39^ While these implementations harbor great potential for applications such as Raman spectroscopy and on-chip quantum communications, none is able to provide the combination of high overall detection efficiency, low dark counts, and parallel time and spectrum detection at single-photon level. The spectroSPAD spectrometer (Figure 2) achieves precisely that by employing a high-performance linear SPAD array as a detector in a Czerny–Turner spectrometer. Whereas a detailed description of the experimental setup is given in Supporting Information Section S1, we provide here a brief account. A microscope with a high numerical aperture objective is used to focus pulsed laser illumination on a single QD, and to collect epi-detected fluorescence. This signal is spectrally filtered from the excitation laser with a dichroic mirror and a dielectric filter (not shown) and imaged by a second lens. This image serves as the input for a spectrometer setup: a 4f system with a blazed grating at the Fourier plane. At the output image plane of the spectrometer, a monolithic linear SPAD array is placed such that each pixel is aligned with the image of a different wavelength range. The detector’s photon detection efficiency (PDE) is ∼8*%* at 620 nm and ∼11*%* at 530 nm (this can be improved, see Discussion) and the median dark count rate is ∼33 counts per second per pixel. We analyze the signal of 40 out of the 512 pixels available in the array, thereby spanning approximately 80 nm around a center wavelength of 620 nm. This results in a spectral resolution of ∼2 nm (6–7 meV). Single-photon detections are time-tagged by an array of 64 time-to-digital converters (TDCs) programmed in the firmware of a field-programmable gate-array (FPGA). Finally, time and wavelength tagged data is analyzed with a dedicated MATLAB script.

Insets (ii) and (iii) show possible visualizations of fluorescence data collected by the system from a single QD, as 2D detection histograms (see Supporting Information Section S2 for QD details). The spectrum over time is seen in (ii), where the time of detection spans the horizontal axis (10 ms time-bins) and energy spans the vertical axis (6–7 meV energy-bins). The effects of thermal broadening, spectral diffusion, and blinking, discussed above, can be clearly observed through the width of the spectral peak at each time-bin (35–50 meV fwhm), the temporal jitter of the spectral peak position, and the variation of emitted intensity, respectively. To achieve spectrally dependent fluorescence decay curves, shown in (iii), the same data set is analyzed by binning the detections according to their delay from the preceding excitation pulse. Note that a full horizontal binning (FHB) of either histogram is equivalent to a standard spectrometer measurement, and a full vertical binning (FVB) to a standard analysis from a single-SPAD measurement.

### 1X Spectral Dynamics

**Figure 3:**
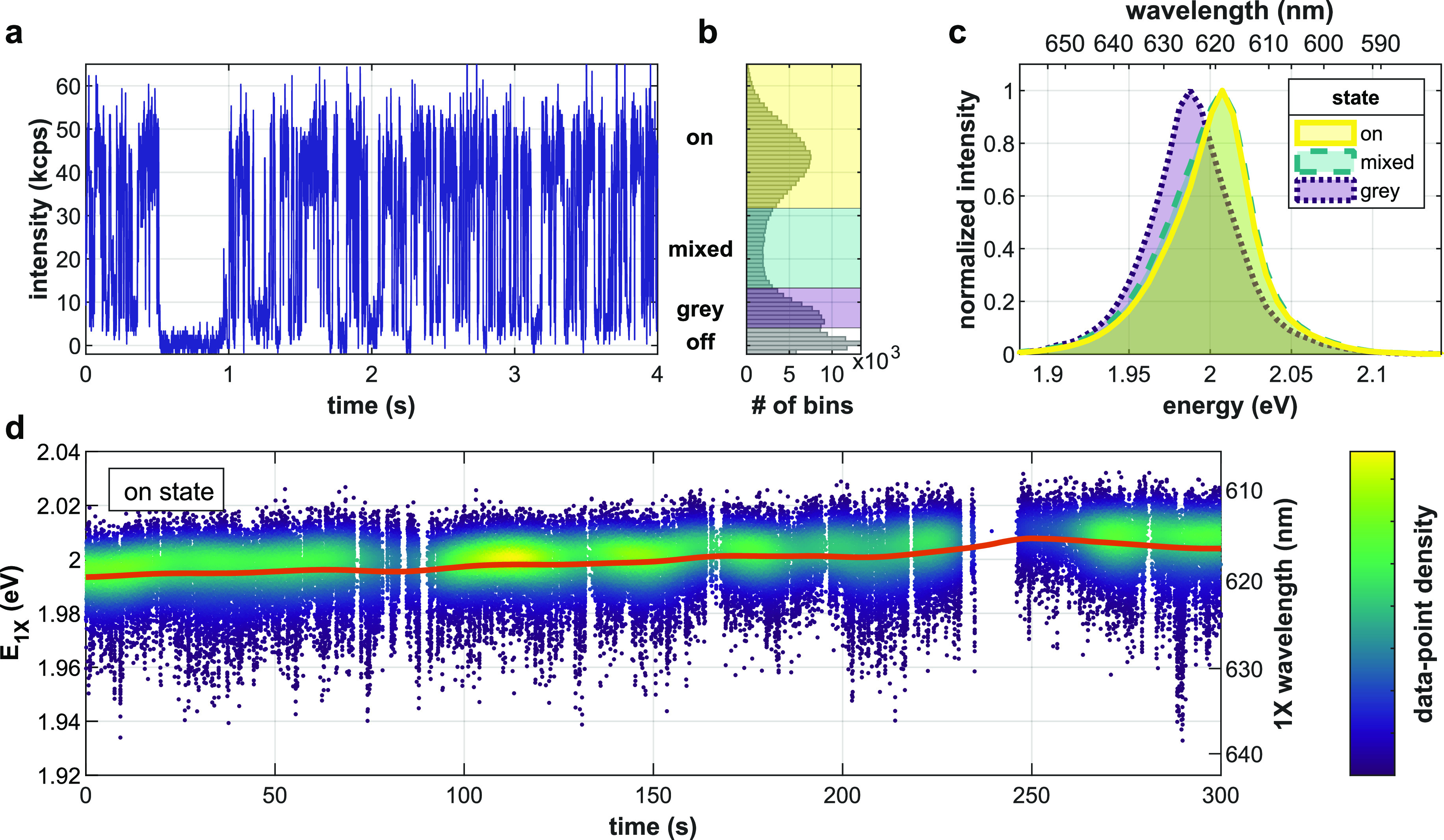
Spectral dynamics of a single QD. (a) Total detected fluorescence intensity, collected over all pixels, versus time in a 4 s period (1 ms time-bins). (b) Histogram of intensity values over a 5 min measurement. Intensity states are marked by colored shading: “off”, “gray”, “mixed”, and “on”. (c) Spectrum according to intensity gating. Note the gray state’s red-shift with respect to the on state. (d) The on state spectral peak evolution over time. Each point is the momentary mean photon energy for a 1 ms time-bin of the on state (⟨*E*⟩_1ms_), colored according to the local density of data-points for clarity. The red line, ⟨*E*⟩_10s_, presents a moving Gaussian-weighted average (σ = 10 s).

Prior to analyzing the BX state, it is important to first study the 1X state. Indeed, simultaneous acquisition of both temporal and spectral data enables a more in-depth analysis of the fluorescence spectral dynamics. Figure 3 employs such an analysis to identify and quantify the spectral broadening effects shown in Figure 1b,d for a single QD measurement. Figure 3a shows the total fluorescence intensity collected over all array pixels at 1 ms time-bins. The intensity trace features a characteristic blinking behavior, that is, stochastic switching between a bright (“on”), dim (“gray”), and dark (“off”) fluorescent states.^40,41^ The presence of these three states is clearly evident in Figure 3b, a histogram of fluorescence intensities over the entire 5 min measurement. Some time-bins are classified as “mixed”, interpreted as time-bins where the QD spent comparable time in different states. Analyzing the spectrum of each intensity state (Figure 3c), reveals a clear red-shift of the gray state spectra, as well as an order-of-magnitude shorter fluorescence lifetime (see Supporting Information Section S3). This supports an identification of the gray state as emission from a charged exciton state (identified as a negative trion in past work^41^). According to ensemble measurements, the BX state is also expected to present a shorter lifetime and shifted emission compared with the on state, making it difficult to differentiate the two contributions.

In addition to blinking, the emission also exhibits spectral diffusion. Figure 3d shows the spectral evolution of the neutral 1X emission (on state) over time. Each dot represents the momentary mean photon energy over a 1 ms time-bin (⟨*E*_1X_⟩_1ms_), colored according to the local density of data-points for clarity. The red line represents a Gaussian-weighted (σ = 10 s) moving average of these values (⟨*E*_1X_⟩_10s_). This smoothed trend shows a gradual wavelength change, predominantly toward shorter wavelengths, possibly due to oxidation.^42^ The fast spectral diffusion dynamics are evident in the distribution of ⟨*E*_1X_⟩_1ms_ around this moving average, Δ*E*_1X_ ≜ ⟨*E*_1X_⟩_1ms_ – ⟨*E*_1X_⟩_10 s_. These faster dynamics are typically attributed to rapid fluctuations in the local electrostatic potential, leading to a shift in emission energy according to the quantum confined Stark effect.^43^

The above results emphasize the difficulty in isolating the BX state emission. Namely, its rather weak contribution is overshadowed by spectral broadening, especially by the gray state emission, which overlaps with it in both spectral and temporal domains. As a result, even a comprehensive analysis of the 2D lifetime-spectrum data (see Supporting Information Section S4) was unable to resolve the BX state spectrum.

### Heralded Spectroscopy

**Figure 4:**
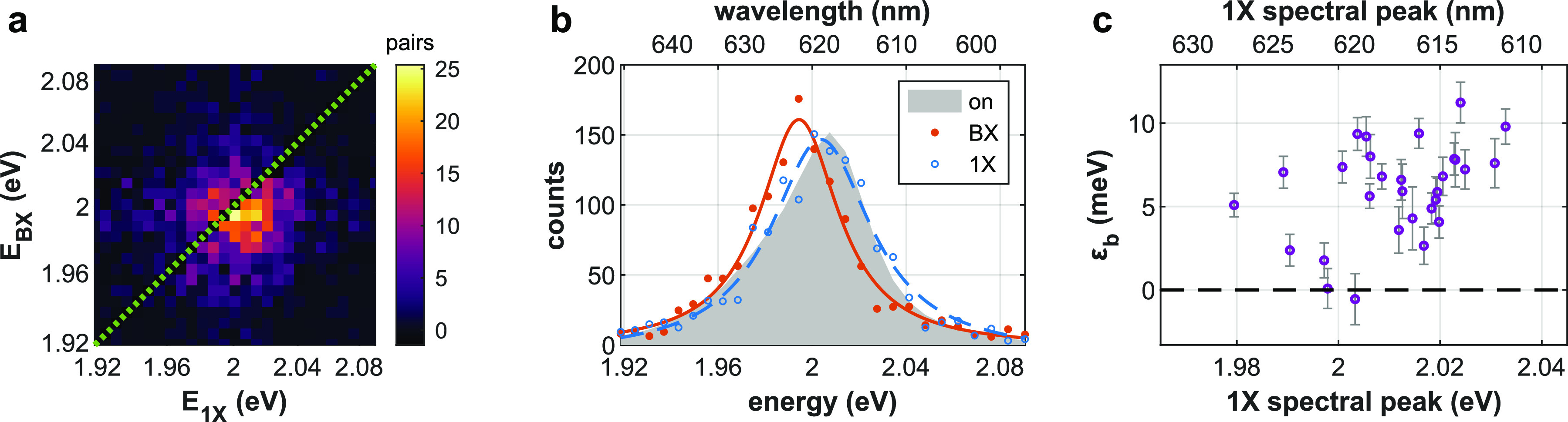
Heralded spectroscopy. (a) Two-dimensional histogram of photon pairs following the same excitation pulse, according to the energy of the first (*E*_BX_) and second (*E*_1X_) photons (vertical and horizontal axes, respectively), over a 5 min measurement. Dashed green line serves as a guide to the eye, marking the same energy for both photons (undetectable by the system). (b) Spectrum of the BX (red dots), 1X (blue rings), and all on state detections (gray area, normalized). Solid red and dashed blue lines are fits of the BX and 1X spectra, respectively, to a Cauchy–Lorentz distribution. Binding energy, estimated as the difference between BX and 1X spectral peaks, is ε_b_ = 9.3 ± 1.0 meV. (c) Binding energy as a function of 1X spectral peak for 30 QDs. Error bars depict 68% confidence intervals.

To directly probe the BX emission, pairs of photon detections following the same excitation pulse are postselected. Such paired events are the result of an excitation to the BX state, and two subsequent radiative relaxations (Figure 1a). We note that due to the low quantum yield of the BX (∼9%, see Supporting Information Section S5 and ref (44)), this is not the most probable route for relaxation from the BX state. Yet, its occurrence provides sufficient signal for our analysis. Applying this postselection (see details in Supporting Information Section S6) to the 5 min single-QD acquisition shown in Figure 3, yields ∼1.4 × 10^3^ pairs over 1.5 × 10^9^ excitation pulses (in agreement with theory, see Supporting Information Section S5). The 2D spectrum of photon pairs, showing the distribution of the energy of the first emitted photon as a function of that of the second, is shown in Figure 4a. The distribution is clearly centered below the diagonal, indicating BX binding. Note that events where both photons of a single cascade impinge on the same pixel are not detected by the system due to pixel dead time (∼25 ns). Figure 4b highlights the first insight that can be derived by such an approach, that is, the BX spectrum (red dots, FHB of panel a) is red-shifted with respect to the 1X spectrum (blue rings, FVB of panel a). The agreement of the 1X spectrum with the overall spectrum of the on state (gray area), corroborates this distinction. The BX binding energy for this particular QD, estimated as the difference between the BX and 1X spectra peaks (extracted by fitting a Cauchy–Lorentz distribution, shown as lines in Figure 4b), is ε_b_ = 9.3 ± 1.0 meV (68% confidence interval).

We note that the identification of the 1X and BX spectral peaks is done here without ambiguity. While previous studies required a power dependence series to correctly assign the 1X and BX states,^16,20^ heralded spectroscopy obviates this requirement. More importantly, this approach super-resolves the few millielectronvolts separated 1X and BX spectral peaks despite their ∼50  meV fwhm and clearly distinguishes between the overlapping BX and gray state emission. In fact, owing to the unprecedented sensitivity of this method, measuring QDs featuring lower ε_b_ than almost all previous measurements of II–VI semiconductor QDs did not incur any additional challenge (see Discussion below).

Thanks to the single-nanocrystal nature of this method, it is not limited to measuring ensemble averaged properties but can also observe their distribution within the ensemble. Figure 4c shows that the BX binding energy increases with the 1X spectral peak position for 30 QDs taken from the same sample. This can be explained as a result of the variation in the physical size of the synthesized QDs. For the QDs investigated in this work, a higher energy 1X spectral peak is likely associated with a thinner CdS shell. A thinner shell also corresponds to further confinement of the electrons in the core and an increased Coulomb interaction between charge carriers, leading to a higher BX binding energy. This trend is in agreement with ensemble measurements for CdSe/CdS seeded nanorods.^27^

### ε_b_ −*E*_1X_ Correlation

**Figure 5:**
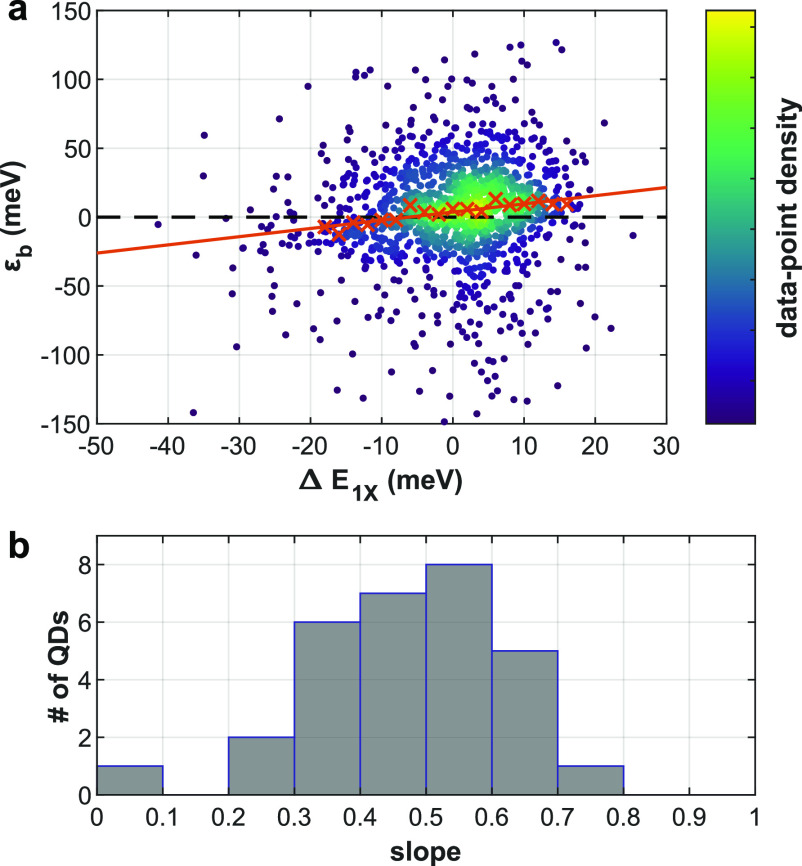
BX binding energy fluctuations are correlated with 1X spectral diffusion. (a) Estimated binding energy (ε_b_) as a function of the momentary 1X spectral shift (Δ*E*_1X_) for a single QD. Each point represents one postselected BX event, colored according to the local density of data-points for clarity. As a guide to the eye, red crosses mark median binding energy for each 2 meV window of Δ*E*_1X_. The red line represents a linear fit to these medians (slope 0.59 ± 0.08). (b) Histogram of the ε_b_ median slope (red line in (a)) for 30 QDs.

Further insight into the BX state can be obtained from comparing the temporal fluctuations of the 1X and BX spectral peaks. As demonstrated in Figure 4, time-resolved heralded spectroscopy enables isolating the BX energy shift despite the spectral fluctuations. Alternatively, one can refer to the 1X spectral position as a sensor for the microenvironment of the nanocrystal, specifically to the fluctuating local electric field, and observe how the BX binding energy reacts to such fluctuations. Figure 5a shows the bivariate distribution of ε_b_ and Δ*E*_1X_, estimated for each postselected BX photon event of a single QD on state measurement. While the distribution of both variables is widened by the various spectral broadening mechanisms discussed above, one can observe a clear correlation between them. As a guide to the eye, we added red crosses to mark the median binding energy for each 2 meV Δ*E*_1X_ window. The red line represents a linear fit of these medians, emphasizing the positive cross-correlation of ε_b_ and Δ*E*_1X_. The slope of this line is 0.59 ± 0.08 (68% confidence interval); that is, for each 2 meV red-shift (blue-shift) of the 1X emission spectral peak the binding energy is lower (higher) by roughly 1.2 meV. Figure 5b, a histogram of the ε_b_ median slope values for 30 QDs, shows that this positive correlation is evident for all QDs measured. This result suggests that the BX binding energy, much like 1X emission, is subject to the quantum confined Stark effect. A higher local field associated with a red-shift of the 1X emission, is correlated with lower BX binding energy (weaker attraction). This can be attributed to the spatial separation of holes and electrons induced by the external field. Notably, at high enough fields the sign of the BX binding energy flips, indicating repulsive interaction of the excitons. This observation agrees with past results on the effect of charge separation in type-II QD heterostructures on the BX binding energy.^45^ Furthermore, it strengthens the assertion that spectral diffusion indeed originates from fluctuations in the local electrostatic potential.

### Discussion

Due to the practical and fundamental importance of the BX state in II–VI semiconductor nanoparticles, extensive experimental work has been dedicated to the estimation of its energy,^5,19,20,27,45−49^ with ε_b_ values spanning negative hundreds to positive tens of millielectronvolts (see Supporting Information Section S7). In core/shell CdSe/CdS nanocrystals, ε_b_ has been shown to transition continuously from positive (attractive) to negative (repulsive) values when tuning the core or shell diameter.^27^ This is indicative of a transition from type-I to type-II or quasi type-II architecture, where electrons and holes are separated to different layers of a heterostructure.^45^ The few millielectronvolt ε_b_ values measured in this work are in good agreement with particles near this transition.

It is worth noting that for such low ε_b_, ensemble measurements become increasingly challenging, as they demand resolving two highly overlapping spectral peaks. Ensemble techniques are, therefore, more readily applied to measure particles exhibiting tens of millielectronvolts BX binding energies, which are indeed reported more often for II–VI nanocrystals (see Supporting Information Section S7). More importantly, ensemble methods require the delicate analysis of a power-series measurement and therefore are prone to systematic biases due to power-dependent charging and absorption cross-section heterogeneity. In fact, such heterogeneity in the sample was suggested as the source of recent controversy regarding the BX binding energy in perovskite nanocrystals.^21,22^ The background-free (isolated BX spectrum) and single-particle nature of heralded spectroscopy afford an unprecedented, sub-millielectronvolt, sensitivity in BX binding energy measurement at room temperature while at the same time circumventing the above-mentioned biases.

While enabling previously inaccessible measurements, at the present there are two limiting factors that should be taken into account when implementing on-chip SPAD arrays in correlative-spectroscopic setups. First, the detection efficiency (∼10% PDE) is still low compared with common camera alternatives. However, this is in part due to the circuit board design implementation used here, dictating signal collection from only every other pixel and a relatively low gain voltage on the diodes. The next system iteration (currently in development), specifically tailored for the purpose of spectroSPAD, is expected to achieve >30% photon detection efficiency, similar to SPAD arrays implemented with the same technology.^44^ This will give rise to an order of magnitude increase in the correlation signal level due to its quadratic dependence on the detection probability. Moreover, detection efficiency is complemented by the very low noise level of state-of-the-art SPAD arrays. Unlike commonly used cameras, these arrays avoid readout noise and feature median dark count rates well below 100 counts per second per pixel.^50^ A second factor to consider, common in detector arrays and specific to photon correlation analysis, is interpixel crosstalk. Due to the close-packing of pixels, a detection in one pixel has a small probability to lead to a false detection in a neighboring pixel, and hence a false photon pair. Any bias due to this effect is mitigated here by a combination of the chip design and temporal gating (see Supporting Information Section S6), bringing the crosstalk probability down to ∼10^–5^, and of a statistical correction as described in ref (44).

## Conclusions

Heralded spectroscopy of BX emission cascades enabled us to perform direct observation and unambiguous identification of emission from multiply excited states of single QDs at room temperature. In addition to avoiding the pitfalls of indirect and ensemble approaches, by separating the BX and 1X emission in the time domain we greatly extend the range of nanomaterials in which we can observe the BX state and allow new insights into exciton–exciton interaction within the single nanoparticles. Our study reveals a positive correlation between exciton–exciton attraction and tighter charge-carrier confinement of the single QDs. We also unveil a fluctuation of this attraction strength, correlated with the fast fluctuations of the local electrostatic potential, and significant enough to lead to exciton–exciton repulsion. These capabilities represent a new tool to probe QD physics and can lead to better design of QD-based technologies where multiexcitonic states typically play a major role.

All this is enabled by constructing the spectroSPAD - a SPAD-based correlative spectrometer extending the temporal resolution limit of standard spectrometers by several orders of magnitude. This tool is not only useful for probing the physics of charge-carrier dynamics but can also address current challenges in quantum optics and communication.

## Supporting Information Available

Details of the spectroSPAD system and the linear SPAD array; details of the quantum dots used in this work including synthesis, sample preparation and excitation saturation estimation; fluorescence decay lifetime by intensity state analysis; 2D lifetime-spectrum analysis; BX quantum yield estimation; heralded spectroscopy analysis and correction details; published values of BX binding energies in II–VI nanocrystals (PDF)
