# Supplementary material for: Heralded Spectroscopy Reveals Exciton–Exciton Correlations in Single Colloidal Quantum Dots
Source: Nano Lett. 2021 Aug 16;21(16):6756–63. doi: 10.1021/acs.nanolett.1c01291 (PMC8397400; doi:10.1021/acs.nanolett.1c01291)
Supplement: Supplementary file 1 — nl1c01291_si_001.pdf [file nl1c01291_si_001.pdf]

Heralded spectroscopy reveals exciton-exciton correlations in single colloidal quantum dots - supporting information

Gur Lubin,[†,§] Ron Tenne,[†,‡,§] Arin Can Ulku,[¶] Ivan Michel Antolovic,[¶] Samuel Burri,[¶] Sean Karg,[†] Venkata Jayasurya Yallapragada,[†] Claudio Bruschini,[¶] Edoardo Charbon,[*,¶] and Dan Oron[*,†]

†*Deptartment of Physics of Complex Systems, Weizmann Institute of Science, Rehovot, Israel*
‡*Department of Physics and Center for Applied Photonics, University of Konstanz, Konstanz, Germany*
¶*School of Engineering, École polytechnique fédérale de Lausanne (EPFL), Neuchâtel, Switzerland*
§*Contributed equally to this work*

E-mail: edoardo.charbon@epfl.ch; dan.oron@weizmann.ac.il

Abstract

This supporting information describes in further detail the data analysis scheme and presents complementary analysis and information to the results described in "Heralded spectroscopy reveals exciton-exciton correlations in single colloidal quantum dots". The sections are brought in the order of reference in the main text: Details of the spectroSPAD system and the linear SPAD array; details of the quantum dots used in this work including synthesis, sample preparation and excitation saturation estimation; fluorescence decay lifetime by intensity state analysis; 2D lifetime-spectrum analysis; BX quantum yield estimation; heralded spectroscopy analysis and correction details; published values of BX binding energies in II-VI nanocrystals.

S1 spectroSPAD system details

This section presents a detailed overview of the experimental apparatus, and further technical details of the linear SPAD array detector at its core.

S1.1 spectroSPAD setup overview

The system is built around a commercial inverted microscope (Eclipse T*i*-U, Nikon). A pulsed diode laser (470 nm, 5 MHz, LDH-P-C-470B, PicoQuant) is focused through an oil immersion objective (x100, 1.3 NA, Nikon) on a single QD. Illumination power density at the sample plane is ~140 W/cm^2 leading to ~66% probability to excite at least one exciton (per pulse, see subsection S2.2). The same objective is used to collect the emitted fluorescence, while back-scattered laser light is filtered by a dichroic mirror (505 LP, Chroma) and a long-pass dielectric filter (488 LP, Semrock). At the output of the microscope, the spectrometer consists of a collimating lens, a blazed grating (235 g/mm, 5.06° blaze, 53-*-790R, Richardson) and an imaging lens, resulting in 3.9×10^{-5} reciprocal linear dispersion and ~6 Å spectral resolution (FWHM). At the spectrometer output image plane, a 512 pixel linear SPAD array (an upgraded version of the sensor described in ref 1, see details in subsection S1.2), is placed such that the active pixel pitch is ~2 nm in wavelength (every second pixel is active). An FPGA with an implemented TDC array (synchronized with the laser) assigns timestamps and pixel addresses to single detections in 40 pixels of the array. The trace of detections is then analyzed by a dedicated MATLAB script, implementing temporal and intensity corrections (see section S6) and the analysis schemes.

S1.2 SPAD array technical details

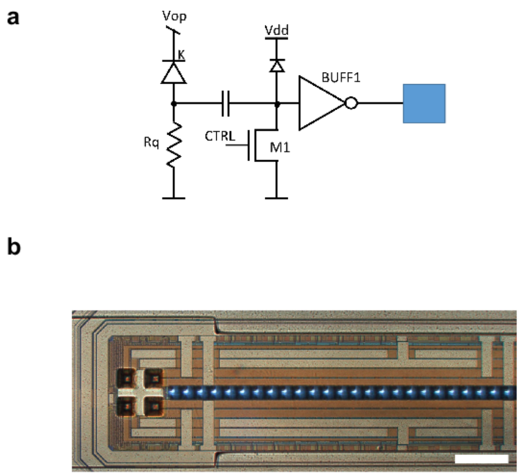

Figure S1: **The linear SPAD array. a)** Electrical circuit of a SPAD array pixel. **b)** Optical image of the detector array, mounted with microlenses. Each blue square represents a single pixel. Scale bar is 100 μm.

Since the creation of the first single-photon avalanche diode (SPAD) in complementary metal-oxide semiconductor (CMOS) in 2003, research in the field of SPADs and SPAD image sensors has led to the creation of the first integrated array in 2004, followed by a wide range of SPAD based image sensors with advanced functionality and continuously increasing speed.

The sensor used in this paper comprises an array of 512 SPAD pixels based on the design proposed in ref 2. Each pixel comprises a SPAD quenched and recharged passively through a poly resistor. The SPAD is interfaced to the exterior of the chip through a circuit described in Figure S1a, including capacitive decoupling, a clamp to V_{dd}, and a low-threshold buffer. The purpose of this circuitry is to ensure low threshold of detection of the avalanche, thus optimizing jitter while controlling noise. Quenching resistor R_q is designed to present a sufficiently high impedance to the anode of the SPAD, while minimizing the avalanche current, so as to control the overall power consumption of the chip. V_{op} is set to $V_{BD} + V_{EX}$, where $V_{BD} \approx 24.4\,\text{V}$ and $V_{EX} \approx 1.6\,\text{V}$ are the breakdown and excess bias voltages, respectively.

The chip was mounted directly on a board with the SPAD outputs wire-bonded and connected to a field-programmable gate array (FPGA), which hosts the TDCs that enable the time characterization of the response. The TDC array is an improved version of the earlier implementation detailed in ref 1. By multiplexing over 64 TDC channels, up to 256 pixels of the array can be temporally correlated. Extending the system to two FPGA boards, enables simultaneous read out from all 512 pixels. However, to reduce DCR, only 40 pixels were used in this work. The DCR reduction is achieved both by collecting DCR from fewer pixels, and by avoiding the few 'hot pixels' in the array that feature exceptionally high DCR (typically 'hot pixels' are defined as those that feature DCR at least two orders of magnitude higher than the median; here the 'noisiest' pixel DCR was just a factor of 3 above the median (104 cps)). The choice of 40 pixels resulted in overall DCR that is about an order of magnitude lower than the detected 'on' state emission in intensity measurements. After temporal gating (see section S6) the number of DCR induced pairs was also about an order of magnitude lower than the total number of detected pairs. Pixels exhibit an average jitter of 105 ps (FWHM) and a median DCR of 33 cps, their native fill factor (without microlenses) and pitch are 25.1% and 26.2µm, respectively. Figure S1b shows a detail of the SPAD array, including microlenses. Microlenses were deposited on the chip to enhance effective fill factor and thus overall photon detection efficiency (PDE).

The recorded temporal response after the TDC (Figure S2) features two peaks: A narrow main peak accounting for ~90% of the counts, and a secondary broader peak delayed by ~3.5 ns, accounting for the remaining ~10%. The presence of the secondary peak is specific to the particular implementation used in this work, and is not generally evident in similar detector arrays. Preliminary results suggest that this irregular temporal response can be eliminated by better firmware design in the next system iteration (currently in development). The QDs investigated in this work featured 1X decay lifetimes significantly longer than this artifact (see section S4). Hence, any ambiguity in order of arrival can be negated by temporal gating (as detailed in section S6) with minimal loss of signal.

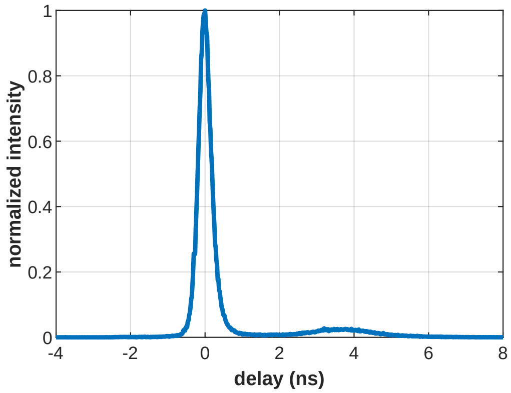

Figure S2: **Temporal response function of a typical pixel.** A pixel of the array is illuminated directly with the synchronized excitation laser (laser pulses are <160 ps FWHM), and recorded through the TDC. Delay values represent the delay from the response peak.

S2 Quantum dots used in this work

This section describes the synthesis of the QDs used in this work, the sample preparation scheme and an estimation of excitation saturation.

S2.1 Quantum dot synthesis and sample preparation

Colloidal CdSe/CdS/ZnS core/shell/shell QDs were synthesized by the following protocol: A cadmium oxide (CdO), n-tetradecylphosphonic acid (TDPA), and 1-octadecene (ODE) mixture was heated to 280°C in a three-neck flask under argon environment. Next, a stock solution of trioctylphosphine selenium (TOPSe) was rapidly injected. The temperature was then reduced to 250°C until the particles reached the desired diameter. A layer-by-layer growth technique in a one-pot synthesis method[3] was used for shell growth of cadmium sulphide (CdS) and zinc sulphide (ZnS). This resulted in spherical QDs with an outer diameter of 5.3 ± 0.6 nm (see Figure S3). Some of the QDs (< 10%) are slightly elongated up to an aspect ratio of 1:1.5. The quantum yield (QY) was measured with an absolute photoluminescence QY spectrometer (Quantaurus-QY, Hamamatsu), and is 90%.

Samples were prepared by spin coating a glass coverslip with a solution of these QDs dispersed in a 3wt% solution of poly(methylmetacrylate) (PMMA) in toluene.

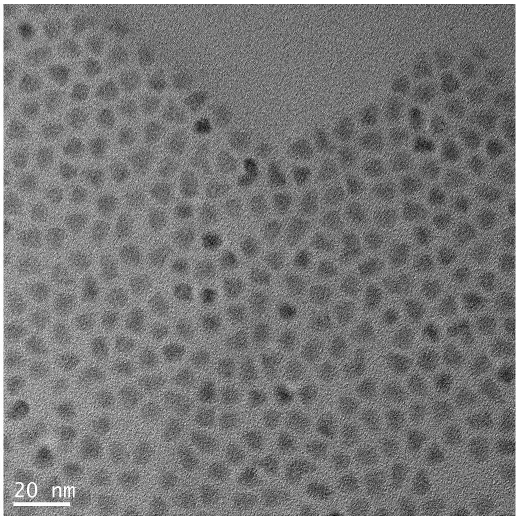

Figure S3: **Transmission electron micrograph of the QDs used in this work.**

S2.2 Quantum dot excitation saturation

To estimate QD excitation saturation, single QDs were illuminated at varying intensities. Figure S4a shows such an experiment, where over time the excitation intensity was increased at a steady rate from 28 W/cm^2 to 280 W/cm^2 and then back down. Higher illumination intensities typically result in more time spent in the 'grey' and 'off' states. Hence, to assess single-excitation saturation, it is essential to identify and estimate the peak occurring intensity of the 'on' state alone. This was achieved by creating an intensity histogram at each value of the excitation power, smoothing the histogram with a Gaussian filter and finding the smoothed histogram peak. The peak occurring values are shown in Figure S4b, together with a fit to a saturation model:[4]

$$P = A \cdot \left(1 - exp^{-\frac{I}{I_{sat}}}\right), \tag{1}$$

where P is the 'on' state peak, I excitation power, I_{sat} saturation power and A asymptotic 'on' state peak (last two are the fit parameters). This simple model assumes negligible contribution to the intensity from multiexcitonic recombination, an assumption justified by the measured value of $g^{(2)}(0) \sim 0.1$ (see Figure S8). The data agrees well with the fit and $I_{sat} = 129 \pm 12$ W/cm^2 (68% confidence interval). The probability to excite n excitons following a single pulse can be estimated from the Poissonian distribution:

$$(n) = \frac{\lambda^n}{n!} e^{-\lambda} \tag{2}$$

using the distribution parameter $\lambda = \frac{I}{I_{sat}}$. At the excitation power used in this work (∼140 W/cm^2, dashed purple line in Figure S4), the probability of exciting at least a single exciton following a laser pulse is ∼66% and of exciting at least twice to form a biexciton ∼30%.

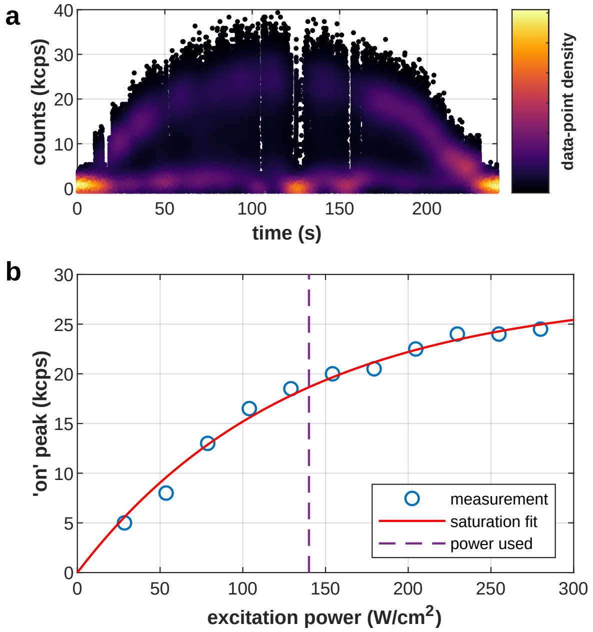

Figure S4: **Quantum dot excitation saturation. a)** Saturation measurement. A single QD illuminated at increasing intensities from 28 W/cm^2 to 280 W/cm^2 and then back down (10 s, ∼25 W/cm^2 steps). Each point represents the detected intensity at a 5 ms time-bin, colored according to the local density of data-points for clarity. **b)** Peak occuring 'on' state intensity for each illumination power (blue circles) and a fit to a saturation curve (red solid line). The excitation power used in this work is marked by a purple dashed line.

S3 Lifetime by intensity state

Figure S5 shows fluorescence decay curves for the different intensity states identified in Figure 3 of the main text. Each trace is a sum over all detector pixels for a specific intensity state. The 'on' and 'grey' state decay traces are both dominated by a single exponential term. However, the 'grey' state decay is an order of magnitude faster ($\tau_{on} \approx 20$ ns and $\tau_{grey} \approx 1.35$ ns, see fit details in next section). An irregular instrument response function (IRF, see subsection S1.2) results in an artifact visible at the first ∼4 ns of each curve.

S4 2D lifetime-spectrum analysis

Apart from heralded spectroscopy, presented in the main text, the single-particle, spectro-temporal information provided by the spectroSPAD can reveal connections between the spectral and the dynamical characteristics of nanocrystal fluorescence. One example of such an observation is presented in the 2D histogram shown

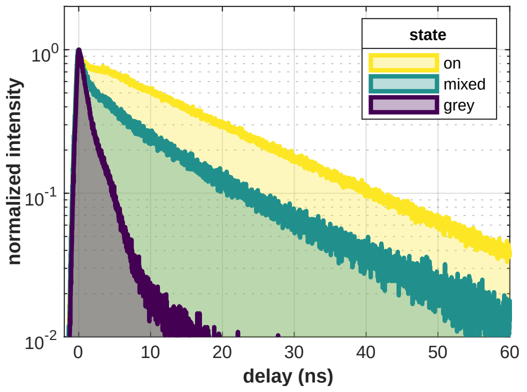

Figure S5: **Fluorescence decay by intensity state.** Histogram of detection delays from the excitation pulse for the intensity states identified in Figure 2 of the main text. Note the different decay lifetime scales of the 'on' and the 'grey' state.

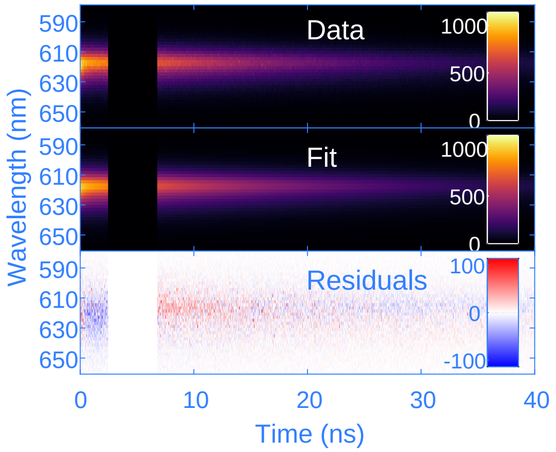

Figure S6: **2D spectrum-lifetime data and fit. Top:** A 2D histogram of photon detections according to their energy (pixel) and arrival time with respect to the excitation laser pulse for the same measurement analyzed in Figures 2-5 of the main text. Here, we include only detections during the 'on' intensity state (see Figure 2 in the main text). **Middle:** A fit of the spectrum-lifetime data. Data in all energies (rows) is fit with the same two exponential terms, allowing the amplitudes to be different for every energy. **Bottom:** residuals between the experimental data and the fit. The missing band of time delays in all data sets were deducted from the fit in order to mitigate the effect of the irregular IRF (see subsection S1.2).

in the top panel of Figure S6: Photon detections (here only from the 'on' blinking state), are binned according to both their arrival time (with respect to excitation pulse) and their energy. In such a spectrum-lifetime dataset, one can differentiate the spectra of different lifetime components. While this type of data is commonly measured for an ensemble of particles (with a scanning monochromator), this is the first demonstration of such a measurement for a single QD.

In the following we attempt to distill the spectrum and lifetime of the biexciton (BX) state from the multi-component data. We show that even when observing the 'on' state data alone, it is extremely difficult to separate the contributions of the charged and the BX state. As a result, we claim that determining the BX binding energy from such an analysis is a challenging task, and is prone to ambiguities.

To analyze the spectrum-lifetime data, we perform a global fit with the same two exponential decaying components for each of the energy-bins (detector pixels). That is, we fit every row in the matrix presented in the top panel of Figure S6, with a sum of two decaying exponential functions. As a result, for n_{pix} pixels, we have $2 + 2{\cdot}n_{pix}$ fit parameters, one lifetime parameter and n_{pix} amplitudes (denoting the spectrum) for each fit component. Note, that in order to reduce the effect of the irregular IRF (see subsection S1.2), we omit a 4.3 ns portion of the data in all fits (the apparent time gap in Figure S6).

Fitting the 'grey' state spectrum-lifetime data (not shown here), we observe that most of the contribution ($> 80\%$) arises from a short lifetime component (1.35 ns) which we associate with the well-known short lifetime of the charged state. The respective spectrum of this state is shown in Figure S7 (purple dots) together with a fit to a Cauchy-Lorentz distribution centered at 1.99 eV (purple line). Next, we turn our focus back to the 'on' state spectrum-lifetime data shown in the top panel of Figure S6. Fitting the data with two decaying exponentials, we obtain a short ($\sim$0.5 ns) and a long ($\sim$20 ns) lifetime components. The long lifetime matches the common lifetime of the single exciton state (1X) in CdSe QDs and indeed its associated spectrum (Figure S7, black dots) resembles that of the 'on' state spectrum presented in figures 3 and 4 of the main text. The peak of a fitted Cauchy-Lorentz distribution (black line) is at 2.007 eV, very close to 2.004 eV obtained from the fit of the heralded 'on' state spectrum.

Unlike the straightforward analysis of the long lifetime contribution, interpreting the short lifetime component is quite a challenging task. First, its integrated intensity is very small, $\sim$1/400 of that of the 1X term. In addition, at short time delays the residuals of the fit (Figure S6, bottom panel), are substantial and systematic (switch from positive to negative sign around 0.4 ns), indicating that our model does not fully account for all features. Most importantly, the spectrum of the short lifetime component (Figure S7, green dots) coincides with that of the charged state.

Considering the intensity proportion of the short lifetime component, the expected contribution to the data

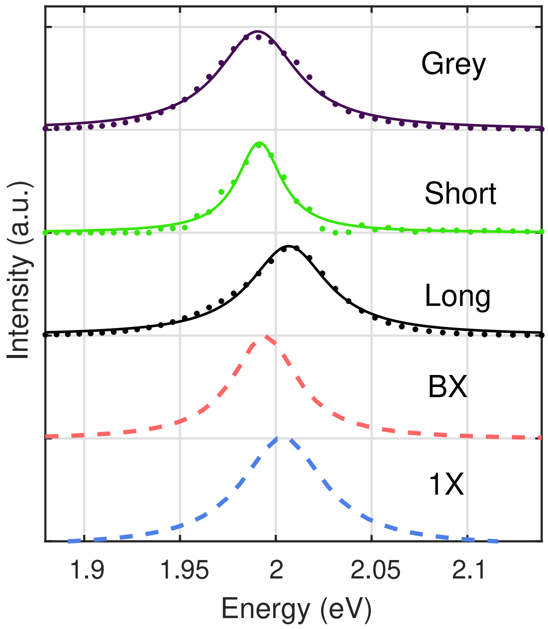

Figure S7: **The spectra of different lifetime components.** Following the fit procedure described in section S4, we obtain a spectrum for each lifetime component separately. The black and green dots show the long and short lifetime spectral contributions to the 'on' intensity state data, respectively. The purple dots show the spectrum of the charged state obtained through the fit of the 'grey' intensity state spectrum-lifetime data. The matching color lines present Cauchy-Lorentz fits for each spectrum. For comparison with the heralded spectroscopy method, described in the main text, we present the fit of the 1X (BX) spectrum for the same QD with a blue (red) dashed line (as shown in Figure 4b of the main text

from BX recombination is given by

$$\frac{N_{2ph}}{N_{1ph}} = \frac{p(N \geq 2)}{p(N \geq 1)} \cdot g^{(2)}(0) \approx 0.05, \tag{3}$$

where $g^{(2)}(0)$ is the second-order correlation function at zero delay time (antibunching factor) and $p(N \geq k)$ is the probability to populate k or more excitons after a single laser pulse, given by a Poisson distribution. In the case of the present measurement, the probability of exciting at least a single exciton was 66% and that of at least two excitons was 30% (see subsection S2.2). The expected calculated intensity proportion is more than an order of magnitude larger than that of the short lifetime fit component.

It is therefore evident that the short lifetime fit component in the 'on' state doesn't match the expected contribution of the BX in both the spectral position and in amplitude. We could not pinpoint the reason for the lack of a fast decaying signal for the BX recombination in the spectrum-lifetime fit. However, we suspect that both the short lifetime of the BX decay and the systematic errors in the fit result in a lack of accuracy for such an analysis. For example, even slight differences in IRF between different pixels can result in a systematic bias that can be overcome only with a complex characterization and modeling procedure.

While a more sophisticated numerical analysis protocol (e.g. taking the IRF into account) may lead to better results, the above analysis shows the challenges of interpreting such a data set. Namely, the contribution of the BX state is extremely weak and its characteristics overlap other states. For example, a fit that includes three decaying exponentials for the 'on' state resulted in over-fitting of the results and the emergence of unnatural spectral features. In contrast, heralded spectroscopy, presented in Figure 4 of the main text, unambiguously isolates the BX component from the 1X and charged 'grey' state, enabling a reliable and robust estimation of the BX spectra.

S5 Biexciton quantum yield

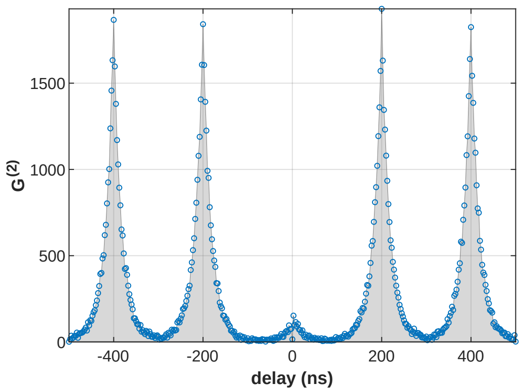

Figure S8: **Second order photon correlations.** Second order photon arrival time correlations extracted from the measurement featured in Figures 3-5 of the main text. The histogram uses 1.25 ns delay bins.

The BX QY to 1X QY ratio can be estimated from the second order correlation of photon arrival times seen in Figure S8. The figure was generated and corrected by applying the method described in ref 5 to the spectroSPAD measurements. Briefly, the measurement described in the main text can be viewed as a multiple arm Hanbury Brown and Twiss correlation setup, where the combination of spectral thermal broadening and the spectrometer are used instead of beamsplitters to multiplex the detection. Second order correlation of arrival times is calculated by histograming delays between detections (arrival times) in different pixels, and temporal and intensity corrections are calculated from the same data used in section S6. The result are peaks separated by the pulse repetition period (200 ns), and broadened by the fluorescence decay lifetime (τ~20 ns). The zero delay peak is significantly lower indicating antibunching

- the lower probability of two photon detections following the same pulse. The ratio of BX QY and 1X QY, is equal to the normalized second order correlation at zero time delay ($g^{(2)}(0)$), calculated as the ratio between the zero delay peak area and the mean area of all the non-zero delay peaks. The value for this specific QD estimated from Figure S8 is $g^{(2)}(0) = 0.09 \pm 0.02$ (standard error). The artifact seen near zero time delay originates from shot noise on the significant crosstalk correction. A thorough study of identical QDs from the same synthesis with a more accurate setup showed that this value is within the expected range of $g^{(2)}(0) = 0.10 \pm 0.02$ (standard deviation).[5] The 1X QY is ∼90% (see section S2), and hence the BX QY is ∼9%.

The ratio between heralded photon pair detections and all detections (α_{BX}), under the excitation conditions used in this work (see subsection S2.2), can be estimated as

$$\alpha_{BX} \sim \frac{N_{2ph}}{N_{1ph}} \sim 0.45 \frac{g^{(2)}(0) \cdot 0.5 \cdot p_{det}^2}{p_{det}} \approx 0.23 \cdot p_{det} \cdot g^{(2)}(0), \quad (4)$$

where p_{det} is the probability to detect a photon per laser pulse from a single QD. The factor of 0.45 is the result of the ratio between of the probability of exciting at least a single exciton (66%) and that of at least two excitons (30%) (see subsection S2.2). During the 'on' state of the QDs measured in this work, values of $p_{det} \sim 10^{-2}$ and $g^{(2)}(0) \sim 10^{-1}$ are measured, resulting in $\alpha_{BX} \sim 2 \cdot 10^{-4}$. This value is in excellent agreement with the α_{BX} value extracted by the heralded approach ($(2.0 \pm 0.7) \cdot 10^{-4}$ for the 30 QDs shown in Figures 4c and 5b of the main text), further corroborating the reliability of these results.

S6 Analysis details

This section details the heralded spectroscopy parameters and the temporal and intensity corrections appended to the acquired data.

S6.1 Heralded spectroscopy

Photon pairs were time gated to support correct identification of BX and 1X emission and reduce the contribution of background due to dark counts. BX emission was gated to the first 5 ns following the excitation pulse. Due to the short lifetime of the BX state, this gating leads to a negligible loss of signal accompanied with a significant reduction of noise. 1X detections were gated to $5 - 60$ ns delay from the BX detection. The upper bound serves to reduce noise while accommodating the longer lifetime of the 1X state emission (see section S4). The lower limit filters out possible misidentification of BX and 1X due to the IRF (see subsection S1.2). The upper limits for BX and 1X also assert that only photon pairs following the same pulse are taken into account. Emission spectral peaks were then estimated by a fit to a Cauchy–Lorentz distribution.

In Figure 4 of the main text, E_{BX} and E_{1X} correspond to the energies of the first and second photons, respectively, of the pairs identified by the time gating described above. In Figure 5a of the main text, the momentary mean 1X energy ($\langle E_{1X} \rangle_{1\,\mathrm{ms}}$) and averaged 1X energy ($\langle E_{1X} \rangle_{10\,\mathrm{s}}$) are estimated at the time of each such pair event (as described in Figure 3d of the main text). $\Delta E_{1X} \triangleq \langle E_{1X} \rangle_{1\,\mathrm{ms}} - \langle E_{1X} \rangle_{10\,\mathrm{s}}$ is used as an estimator of the momentary spectral shift (horizontal axis). The momentary BX binding energy is estimated as the difference between $\langle E_{1X} \rangle_{1\,\mathrm{ms}}$ and E_{BX} (vertical axis). Using these estimators (i.e. $\langle E_{1X} \rangle_{1\,\mathrm{ms}}$ instead of E_{1X}) is beneficial when estimating momentary spectral shift and binding energy for each pair event separately, as the 1 ms averaging averages over the thermal broadening distribution of 1X emission, reducing its effect on the final results.

S6.2 Temporal Corrections

The time-to-digital converter (TDC) architecture assigns timestamps with a mean interval of ∼18 ps (the detector jitter is larger, see subsection S1.2). However, as detailed elsewhere,[1] the timestamps are not uniformly spaced but rather each span a $0 - 92$ ps range of arrival times (most spans are within 18 ± 12 ps). This non-uniformity was characterized by illuminating the detector with temporally featureless halogen light, and recording the occurrence of each timestamp as a measure of the relative time duration it spans. The correction was then implemented statistically by assigning to each recorded raw-timestamp a corrected-timestamp chosen at random from the respective time span. In addition, timestamps recorded for each detector pixel are differently delayed from the TDC trigger. This was characterized by illuminating the detector directly with the <160 ps FWHM excitation laser pulse, and adding a per-pixel timestamp delay so that the recorded pulse peaks in all detectors temporally overlap.

S6.3 Intensity corrections

Two sources of false detections and detection pairs had to be considered in the analysis of heralded spectroscopy. The first, dark count rate (DCR), was recorded and subtracted from the intensity trace (per pixel). The expected number of DCR-photon detection pairs was estimated and subtracted from the photon pair signal (DCR-DCR pair occurrence is negligible). The second source of false photon pairs, detector crosstalk, was characterized and corrected statistically, by the protocol detailed in ref 5.

In Figure 5a of the main text, each data-point represents a single photon pair event. Hence, only in this figure, intensity corrections could not be implemented with this statistical approach. The number of DCR and crosstalk induced pairs in this figure can be estimated to

be ~10% of the overall data-points. Hence, the use of a median estimator (red crosses in Figure 5a of the main text) mitigates the effect of noise induced outliers. Furthermore, to avoid biases where noise might be more significant than signal, the median values shown and considered for the fit were only for ΔE_{1X} energy-bins including at least 1% of the total signal.

All corrections were verified to be stable over time.

S7 Published values of BX binding energy

In order to compare the measured value of BX binding energy in this work to previously reported values in II-VI nanoparticles we have summarized a survey of the literature in Table S1. We note that different works use opposite conventions with respect to the definition of sign in biexciton binding energy. Here, we adopt the convention in which a positive binding energy ($\varepsilon_b > 0$) refers to a BX spectral peak at a lower energy with respect to the 1X spectral peak due to attractive interaction between the excitons. In the case of single particle techniques, such as the current work, we note only the ensemble average BX binding energy in the table.

Table S1: Published values for BX binding energy in II-VI nanocrystals

Material (core/shell)	Shape	Size (radius in nm)	Method*	Year	BX binding energy (meV)	Ref
CdSe/ZnS	dots	1-3.5 core	uPL	2003	**10 - 30**	6
CdSe	dots	2.4-4	uPL	2005	**28 - 38**	7
CdSe/ZnS	dots	1.25-4.5 core	TRPL	2006	**20 - 50**	8
CdTe/CdSe and CdSe	dots	1.9/0-2.5 core/shell	TRPL	2007	**-30 - 30**	9
CdS/ZnSe	dots	1.6/2 core/shell	TRPL	2007	**-106**	10
CdSe	dots	1.8-3	TA	2008	**18 - 25**	11
CdSe:Te doped	dots	1-2	TRPL	2008	**-300 - -100**	12
CdSe	dots	1.5-2.8	TA	2009	**9 - 18**	13
CdSe	dots	1.5-2.8	uPL	2009	**37 - 50**	13
CdSe/CdS	rods	1.1-2/(2x50)	TRPL	2009	**-40 - 30**	14
CdS/ZnSe	dots	1.9	TRPL	2010	**-70 - -20**	15
CdSe/CdS	giant dots	3/8	CS	2010	**-25**	16
CdSe	dots	2.8	TA	2011	**10**	17
CdSe/ZnS	rods	-	CS	2011	**12**	18
CdSe	nanoplatelets	0.67x5x17	TGPL	2019	**45**	19
CdSeS/ZnS	dots	3 core+shell	2DFS	2021	**16**	20
CdSe	nanoplatelets	0.6x3.5x10.5	CS	2021	**16.5**	21
CdSe/CdS/ZnS	dots	5.3 core+shell	HS	2021	**6**	This work

* **uPL** - PL upconversion, **TRPL** - Time resolved PL, **TA** - Transient absorption, **CS** - Single particle PL spectroscopy at cryogenic temperatures, **TGPL** - Transient grating PL, **2DFS** - Two dimensional fluorescence spectroscopy, **HS** - Heralded spectroscopy.

References

(1) Burri, S.; Bruschini, C.; Charbon, E. LinoSPAD: A Compact Linear SPAD Camera System with 64 FPGA-Based TDC Modules for Versatile 50 ps Resolution Time-Resolved Imaging. *Instruments* **2017**, *1*, 6.

(2) Veerappan, C.; Charbon, E. A Low Dark Count p-i-n Diode Based SPAD in CMOS Technology. *IEEE Transactions on Electron Devices* **2016**, *63*, 65–71.

(3) Li, J. J.; Wang, Y. A.; Guo, W.; Keay, J. C.; Mishima, T. D.; Johnson, M. B.; Peng, X. Large-scale synthesis of nearly monodisperse CdSe/CdS core/shell nanocrystals using air-stable reagents via successive ion layer adsorption and reaction. *Journal of the American Chemical Society* **2003**, *125*, 12567–12575.

(4) Teitelboim, A.; Oron, D. Broadband Near-Infrared to Visible Upconversion in Quantum Dot–Quantum Well Heterostructures. *ACS Nano* **2016**, *10*, 446–452.

(5) Lubin, G.; Tenne, R.; Michel Antolovic, I.; Charbon, E.; Bruschini, C.; Oron, D. Quantum correlation measurement with single photon avalanche diode arrays. *Optics Express* **2019**, *27*, 32863.

(6) Achermann, M.; Hollingsworth, J. A.; Klimov, V. I. Multiexcitons confined within a subexcitonic volume: Spectroscopic and dynamical signatures of neutral and charged biexcitons in ultrasmall semiconductor nanocrystals. *Physical Review B - Condensed Matter and Materials Physics* **2003**, *68*, 245302.

(7) Bonati, C.; Mohamed, M. B.; Tonti, D.; Zgrablic, G.; Haacke, S.; van Mourik, F.; Chergui, M. Spectral and dynamical characterization of multiexcitons in colloidal CdSe semiconductor quantum dots. *Physical Review B* **2005**, *71*, 205317.

(8) Oron, D.; Kazes, M.; Shweky, I.; Banin, U. Multiexciton spectroscopy of semiconductor nanocrystals under quasi-continuous-wave optical pumping. *Physical Review B* **2006**, *74*, 115333.

(9) Oron, D.; Kazes, M.; Banin, U. Multiexcitons in type-II colloidal semiconductor quantum dots. *Physical Review B* **2007**, *75*, 035330.

(10) Klimov, V. I.; Ivanov, S. A.; Nanda, J.; Achermann, M.; Bezel, I.; McGuire, J. A.; Piryatinski, A. Single-exciton optical gain in semiconductor nanocrystals. *Nature* **2007**, *447*, 441–446.

(11) Sewall, S. L.; Cooney, R. R.; Anderson, K. E.; Dias, E. A.; Sagar, D. M.; Kambhampati, P. State-resolved studies of biexcitons and surface trapping dynamics in semiconductor quantum dots. *Journal of Chemical Physics* **2008**, *129*, 84701.

(12) Avidan, A.; Oron, D. Large Blue Shift of the Biexciton State in Tellurium Doped CdSe Colloidal Quantum Dots. *Nano Letters* **2008**, *8*, 2384–2387.

(13) Sewall, S. L.; Franceschetti, A.; Cooney, R. R.; Zunger, A.; Kambhampati, P. Direct observation of the structure of band-edge biexcitons in colloidal semiconductor CdSe quantum dots. *Physical Review B - Condensed Matter and Materials Physics* **2009**, *80*, 081310.

(14) Sitt, A.; Sala, F. D.; Menagen, G.; Banin, U. Multiexciton Engineering in Seeded Core/Shell Nanorods: Transfer from Type-I to Quasi-type-II Regimes. *Nano Letters* **2009**, *9*, 3470–3476.

(15) Ivanov, S. A.; Achermann, M. Spectral and Dynamic Properties of Excitons and Biexcitons in Type-II Semiconductor Nanocrystals. *ACS Nano* **2010**, *4*, 5994–6000.

(16) Htoon, H.; Malko, A. V.; Bussian, D.; Vela, J.; Chen, Y.; Hollingsworth, J. A.; Klimov, V. I. Highly emissive multiexcitons in steady-state photoluminescence of individual "giant" CdSe/CdS Core/Shell nanocrystals. *Nano letters* **2010**, *10*, 2401–7.

(17) Sewall, S. L.; Cooney, R. R.; Dias, E. A.; Tyagi, P.; Kambhampati, P. State-resolved observation in real time of the structural dynamics of multiexcitons in semiconductor nanocrystals. *Physical Review B* **2011**, *84*, 235304.

(18) Louyer, Y.; Biadala, L.; Trebbia, J.-B.; Fernée, M. J.; Tamarat, P.; Lounis, B. Efficient biexciton emission in elongated CdSe/ZnS nanocrystals. *Nano letters* **2011**, *11*, 4370–5.

(19) Geiregat, P.; Tomar, R.; Chen, K.; Singh, S.; Hodgkiss, J. M.; Hens, Z. Thermodynamic Equilibrium between Excitons and Excitonic Molecules Dictates Optical Gain in Colloidal CdSe Quantum Wells. *The Journal of Physical Chemistry Letters* **2019**, *10*, 3637–3644.

(20) Mueller, S.; Lüttig, J.; Brenneis, L.; Oron, D.; Brixner, T. Observing Multiexciton Correlations in Colloidal Semiconductor Quantum Dots via Multiple-Quantum Two-Dimensional Fluorescence Spectroscopy. *ACS Nano* **2021**, *15*, 4647–4657.

(21) Peng, L.; Cho, W.; Zhang, X.; Talapin, D.; Ma, X. Observation of biexciton emission from single semiconductor nanoplatelets. *Physical Review Materials* **2021**, *5*, L051601.
